# Dehydroepiandrosterone Ameliorates Abnormal Mitochondrial Dynamics and Mitophagy of Cumulus Cells in Poor Ovarian Responders

**DOI:** 10.3390/jcm7100293

**Published:** 2018-09-20

**Authors:** Chia-Jung Li, San-Nung Chen, Li-Te Lin, Chyi-Uei Chern, Peng-Hui Wang, Zhi-Hong Wen, Kuan-Hao Tsui

**Affiliations:** 1 Research Assistant Center, Show Chwan Health Memorial Hospital, Changhua 500, Taiwan; nigel6761@gmail.com; 2 Department of Medical Research, Chang Bing Show Chwan Health Memorial Hospital, Changhua 515, Taiwan; 3 Department of Obstetrics and Gynecology, Kaohsiung Veterans General Hospital, Kaohsiung 813, Taiwan; snchen@vghks.gov.tw (S.-N.C.); litelin1982@gmail.com (L.-T.L.); cuchern@vghks.gov.tw (C.-U.C.); 4 Institute of Clinical Medicine, National Yang-Ming University, Taipei 112, Taiwan; phwang@vghtpe.gov.tw; 5 Department of Biological Science, National Sun Yat-sen University, Kaohsiung 804, Taiwan; 6 Department of Obstetrics and Gynecology, Taipei Veterans General Hospital, Taipei 112, Taiwan; 7 Department of Medical Research, China Medical University Hospital, Taichung 404, Taiwan; 8 Department of Marine Biotechnology and Resources, National Sun Yat-sen University, Kaohsiung 804, Taiwan; wzh@mail.nsysu.edu.tw; 9 Marine Biomedical Laboratory and Center for Translational Biopharmaceuticals, Department of Marine Biotechnology and Resources, National Sun Yat-sen University, Kaohsiung 804, Taiwan; 10 Department of Pharmacy and Master Program, College of Pharmacy and Health Care, Tajen University, Pingtung County 813, Taiwan

**Keywords:** dehydroepiandrosterone, mitochondrial dynamics, mitophagy, cumulus cells, poor ovarian responders

## Abstract

Mitochondrial dysfunction is related to reproductive decline in humans, with consequences for in vitro fertilization (IVF). We assessed whether dehydroepiandrosterone (DHEA) could regulate mitochondrial homeostasis and mitophagy of cumulus cells (CCs) in poor ovarian responders (PORs). A total of 66 women who underwent IVF treatment at the Reproductive Medicine Center of Kaohsiung Veterans General Hospital were included in this study. Twenty-eight normal ovarian responders (NOR) and 38 PORs were enrolled. PORs were assigned to receive DHEA supplementation (*n* = 19) or not (*n* = 19) before IVF cycles. DHEA prevents mitochondrial dysfunction by decreasing the activation of *DNM1L* and *MFF*, and increasing *MFN1* expression. Downregulation of *PINK1* and *PRKN* occurred after DHEA treatment, along with increased lysosome formation. DHEA not only promoted mitochondrial mass but also improved mitochondrial homeostasis and dynamics in the CCs of POR. We also observed effects of alterations in mRNAs known to regulate mitochondrial dynamics and mitophagy in the CCs of POR. DHEA may prevent mitochondrial dysfunction through regulating mitochondrial homeostasis and mitophagy.

## 1. Introduction

Poor ovarian responders (PORs), characterized by a poor response to controlled ovarian stimulation (COS), pose a great obstacle for in vitro fertilization (IVF). Assisted reproductive technology (ART) is currently a popular technique, and its adoption is increasing [1]. Improving reproductive outcomes of PORs is one of the pivotal issues in ART. Most mammalian females, including humans, experience reproductive decline with age. The fertilization ability and developmental competence of human embryos appear to be directly related to the metabolic capacity of their mitochondria. Mitochondrial dysfunctions resulting from a variety of intrinsic and extrinsic influences, including genetic abnormalities, hypoxia, and oxidative stress, can profoundly deplete the level of ATP generation in oocytes which, in turn, may result in aberrant chromosome segregation or developmental arrest [2].

Our previous studies demonstrated that mitochondrial function was significantly impaired in cumulus cells (CCs) from the POR group, and in human granulosa cells in vitro from the serum free group compared with the control group [3,4]. Hence, considering the importance of mitochondrial function in the oocyte, it is not surprising that oocyte quality decreases with increasing mitochondrial dysfunction [5]. This, in turn, may translate into a decrease in the success rates of IVF cases, partially due to increases in oocyte mitochondrial defects. As mitochondria are involved in oocyte growth and embryo development, interference with mitochondrial function contributes to the arrest of oocyte maturation, impaired fertilization, and compromised embryo development [6,7].

Several strategies, including a variety of COS protocols and various adjuvant supplements, have been proposed to improve the reproductive outcomes in PORs [8,9,10]. While some adjuvants show more promise than others do, to date, none have significantly improved pregnancy rates [11,12]. DHEA is a mild and therapeutically well-tolerated male hormone. It is produced in an intermediate step of steroidogenesis by adrenals and ovaries during the conversion of cholesterol to the two sex hormones, estradiol and testosterone [12,13]. In the female body, DHEA is converted into androgens, mainly testosterone [14,15]. DHEA improves embryo quality and pregnancy chances in PORs [16]. How these effects are achieved is, however, unknown. A small pilot study of limited power suggested that DHEA may reduce aneuploidy [15]. Since aneuploidy in human embryos is frequent and increases with advancing female age, a reduction in aneuploidy could, at least partially, explain improved embryo quality and pregnancy rates.

Improvement in diminished ovarian reserves is a pivotal step in ART. DHEA improves oocyte quality, fertilization rate, embryo quality, and pregnancy rate in PORs. Our previous study indicated that DHEA supplementation for PORs led to significantly decreased apoptosis and decelerated aging in CCs [3,17]. Several studies show that mitochondria also play a key role in oocyte maturation [6,18]. Therefore, the aim of this study was to investigate whether DHEA supplementation can decrease imbalances in mitochondrial dynamics and maintain mitochondrial homeostasis in CCs of PORs. We compared clinical outcomes, mitochondrial dynamics, and the expression of mitophagy genes in CCs between PORs, with or without DHEA.

## 2. Materials and Methods

### 2.1. Study Population

**Figure 1:**
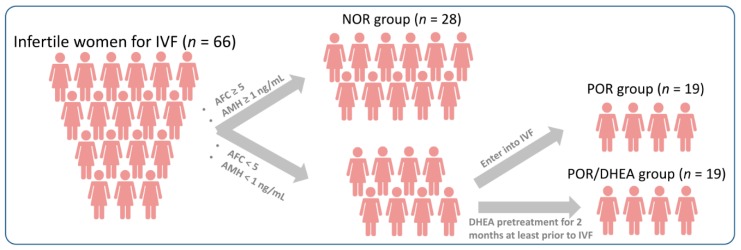
Flow diagram for the selection of eligible studies and subjects.

The infertile women, including normal ovarian responders (NORs) and PORs, who underwent the IVF cycles, were enrolled in this study. The inclusive criteria for NORs were as follows: (1) antral follicle counts (AFC) ≥ 5 or anti-Müllerian hormone (AMH) ≥ 1 ng/mL, and (2) the number of retrieved oocytes was between 4 and 15. PORs met the Bologna criteria, having at least two of the three following features: (1) advanced maternal age (≥40 years) or any other risk factor for POR, (2) a previous POR (≤3 oocytes with a conventional stimulation protocol), and (3) an abnormal ovarian reserve test. An abnormal ovarian reserve test was defined as AFC < 5 or AMH < 1 ng/mL, in this study. Moreover, two episodes of a previous POR after maximal stimulation alone would be sufficient to define a patient as a POR. Patients were excluded if they had a history of previous oophorectomy, underwent a donor cycle, ever received pelvic irradiation or chemotherapy, or took hormonal treatment or DHEA supplementation in recent three months. Informed consent was obtained from all participants after a detailed explanation (Figure 1). All procedures performed in this study were approved by the institutional review board at Kaohsiung Veterans General Hospital (VGHKS106-129; VGHKS107-142).

### 2.2. Study Design

**Figure 2:**
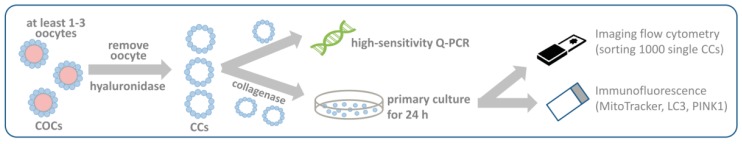
Flow chart of experimental design.

This prospective cohort study was conducted from June 2017 to February 2018 in the Reproductive Center of the Kaohsiung Veterans General Hospital. PORs were divided into the POR group and the POR/DHEA group according to whether they received DHEA (CPH; Formulation Technology, Oakdale, CA, USA) treatment prior to the IVF cycles. In the POR group (*n* = 19), patients directly underwent an IVF cycle without DHEA supplementation. In the POR/DHEA group (*n* = 19), patients were administered a 30 mg DHEA capsule three times daily for at least 8 weeks before entering an IVF cycle. The cumulus oocyte complex (COC) from all patients were collected for further researches (Figure 2). This study was registered with the Clinical Trial Registry (Identifier: NCT03438812).

### 2.3. Treatment Protocol

The gonadotropin-releasing hormone (GnRH) antagonist protocol were applied for all participants. Exogenous gonadotropins, including Gonal-F (rFSH, Merck KGaA, Darmstadt, Germany), Pergovaris (rFSH + rLH, Merck Serono, Aubonne, Switzerland) or Merional (rFSH + rLH, Institut Biochimique SA, Lamone, Switzerland) were administered from the menstrual cycle day 2 or day 3 to the maturity of follicles. GnRH antagonists (Cetrotide 0.25 mg; Merck Serono, Idron, France) were initiated when the leading follicle reached a diameter of 12−14 mm until the day of oocyte trigger. Dual trigger with recombinant human chorionic gonadotropin (rHCG) (Ovidrel, Merck Serono, Modugno, Italy) and GnRH agonist (Lupro, Nang Kuang Pharmaceutical CO, Ltd., Tainan, Taiwan) was administered until at least three dominant follicles reached ≥17 mm in diameter in the NORs, or at least one dominant follicle reached ≥17 mm in diameter in the PORs. Ultrasound-guided oocyte aspiration was performed transvaginally 34−36 h after oocyte trigger. Intracytoplasmic sperm injection (ICSI) was conducted for all PORs to avoid the possibility of total fertilization failure. However, the indication of ICSI for NORs was severe male infertility. Successful fertilization was determined if two clear pronuclei were observed after 18−20 h of insemination. Embryos were evaluated and graded based on the criteria established by the Istanbul consensus workshop. All embryos were frozen by vitrification on the third day after oocyte retrieval.

An artificial cycle was used for endometrial preparation in frozen embryo transfer. Embryo transfer was carried out under the guidance of transabdominal sonography. Daily progesterone with Crinone 8% gel (Merck Serono, Hertfordshire, UK) plus Duphaston 40 mg (Abbott, Olst, The Netherlands) were administered for luteal phase support. Once a biochemical pregnancy was proven by the serum hCG assay 15−16 days after the embryo transfer, daily progesterone was continued for additional 6 weeks. Clinical pregnancy was established when a visible fetal heart beat was observed in an intrauterine gestational sac by transvaginal ultrasound. Ongoing pregnancy was determined by the presence of a fetal heart beat beyond 20 weeks of gestation. Live birth was defined as delivery of a live fetus after 20 completed weeks of gestation.

### 2.4. Collection of CCs

COCs were collected, washed, and incubated in the IVF medium covered with paraffin oil after oocyte aspiration. Cumulus cells were exposed to 40 IU/mL hyaluronidase (SynVitro™ Hyadase, Origo, Målov Denmark, Knardrupvej) for 30 s before being washed with phosphate buffered saline. Then, CCs were isolated and pooled per patient. After oocyte removal, cumulus cells were mechanically disaggregated, pooled, and washed three times through a series of centrifugations at 800 g for 5 min in D-PBS/BSA. The final pellets were resuspended in Histopaque 1077 (Sigma, St. Louis, MO, USA) supplemented with 10% fetal bovine serum (Gibco, Thermo Fisher Scientific, Waltham, MT, USA), 5 mg/L insulin, 5 mg/L transferrin, and 5 μg/L sodium selenite (ITS, Sigma, St. Louis, MO, USA), and 1.25 μM androstenedione (4-androstene-3,17-dione, Sigma). After evaluating the viability of the cells with Trypan blue staining (0.2% *w*/*v*, Sigma, St. Louis, MO, USA), cumulus cells were plated in a 4 well plate (Thermo Fisher Scientific) at a concentration of 2 × 10^4^ viable cells/well (5 × 10^4^ viable cells/mL) to be cultured at 37.5 °C in a humidified incubator at 5% CO_2_ for up to 24 h, for further study (Figure 2).

### 2.5. Mitochondrial Mass Measurement 

The immunoassay was performed as previously described [3]. For the image cytometry assay, the cell pellet was gently and thoroughly resuspended in the remaining 20 μL of media, and transferred to the disposable counting slide. Fluorescent images of the sample were captured using the filter optics module VB-535-402 (Nexcelom Bioscience, Lawrence, MA, USA) for MitoTracker green (Molecular Probes Inc., Eugene, OR, USA) detection at an exposure time of 1 s with a size cut-off of 0.1 μm. One thousand single cumulus cells were obtained, and DAPI blue fluorescence and green fluorescence in the blue channel were collected for quantification.

### 2.6. RNA Extraction and Real-Time PCR

The RNA expression assay was performed as previously described [19]. Total RNA was extracted using TRIzol reagent (Invitrogen, Carlsbad, CA, USA) according to the manufacturer’s instructions. To detect mRNA expression, real-time quantitative RT-PCR (qRT-PCR) analysis was performed using an ABI Prism 7700 Sequence Detection System (Perkin-Elmer Applied Biosystems, Norwalk, CT, USA). PCR was performed using the SYBR Green PCR Core Reagents kit (Perkin-Elmer Applied Biosystems, Norwalk, CT, USA). The thermal cycling conditions comprised an initial denaturation step at 95 °C for 10 min, 40 cycles at 95 °C for 15 s, and 60 °C for 1 min. Experiments were performed with duplicates for each data point. All of the samples with a coefficient of variation for Ct value > 1% were retested. Sequences of the primers used for qRT-PCR assays are shown in Supplemental Table S1.

### 2.7. Immunofluorescence Labeling Assay

The immunoassay was performed as previously described [20]. Cells were incubated with MitoTracker deep red (Molecular Probes Inc., Eugene, OR, USA) at 37 °C for 30 min. To determine the subcellular localization of LC3 (ab48394, abcam) and PINK1 (ab216114), cells were fixed with 4% paraformaldehyde and subsequently permeabilized with 0.2% Triton X-100 on ice for 5 min. After washing with PBS twice, cells were incubated in blocking solution (PBS containing 20% goat serum) at room temperature for 30 min and incubated with primary antibodies at 4 °C overnight. After washing with PBS, the cells were then stained with FITC-conjugated secondary antibodies (1:250, Code 711-545-152, Jackson Immuno Research Lab, Inc., West Grove, PA, USA), counterstained with Hoechst 33258 for 10 min, and visualized by confocal laser scanning microscopy (C1-Si, Nikon Instruments Inc., Tokyo, Japan).

### 2.8. Ethics Statement

This study was approved by the institutional review board of Kaohsiung Veterans General and was performed according to approved guidelines. All participants signed an informed consent. This study was implemented incompliance with the Declaration of Helsinki.

### 2.9. Statistical Analysis

Each experiment was performed at least 6 times, and all data are represented as means ± error mean (SEM) of quadruplicate measurements. The intensity of fluorescence was quantified and analyzed using ImageJ software (NIH), Zen lite software (Carl Zeiss Co. Ltd., Oberkochem, Germany) and MicroP software [21]. Statistical significance was evaluated using two-way analysis of variance test using GraphPad Prism, version 6.0 (GraphPad Software, CA, USA), followed by Tukey’s multiple range test (MRT), to assess differences between group means. Differences were considered significant when *p* < 0.05.

## 3. Results

### 3.1. Basic Characteristics of Patients Undergoing Ivf Cycles

Table 1 shows the baseline features of the NOR, POR, and POR/DHEA groups. A total of 66 women (28 NOR, 19 POR, and 19 POR/DHEA) undergoing IVF cycles participated in this study. Basic demographic characteristics, such as body mass index, duration of infertility, types of infertility, and basal FSH, E2, LH were not significantly different among the three groups. However, members of the NOR group were significantly younger than those of the POR group (36.2 ± 3.0 years vs. 40.5 ± 4.3 years, *p* < 0.05). Furthermore, serum levels of AMH, as well as AFC, were markedly higher in the NOR group than in the POR and POR/DHEA groups. Compared to the POR group, members of the POR/DHEA group had more previous failed IVF cycles (3.5 ± 2.6 vs. 1.7 ± 2.2, *p* < 0.05).

### 3.2. Cycle Characteristics and Clinical Outcomes of Patients Undergoing IVF Cycles

Characteristics of IVF cycles and pregnancy outcome among the NOR, POR, and POR/DHEA groups are shown in Table 2. Regarding stimulation duration and total gonadotropin dose, there were no significant differences among the three groups. Compared with the POR group, the NOR group had significantly higher numbers of retrieved oocytes, metaphase II oocytes, fertilized oocytes, day 3 embryos, and top-quality embryos at day 3. Likewise, the clinical pregnancy rate, ongoing pregnancy rate, and live birth rate were significantly higher in the NOR group than in the POR group.

In the POR/DHEA group, the number of retrieved oocytes (4.1 ± 3.0 vs. 3.0 ± 1.9), metaphase II oocytes (2.0 ± 1.3 vs. 1.6 ± 1.5), fertilized oocytes (2.6 ± 1.6 vs. 2.3 ± 1.7), clinical pregnancy rate (26.3% vs. 11.1%), ongoing pregnancy rate (26.3% vs. 11.1%), and live birth rate (16.7% vs. 11.1%) were increased when compared to the POR group, but the differences were not significant.

### 3.3. Effects of DHEA Supplementation on Cumulus Cell Mitochondria

**Figure 3:**
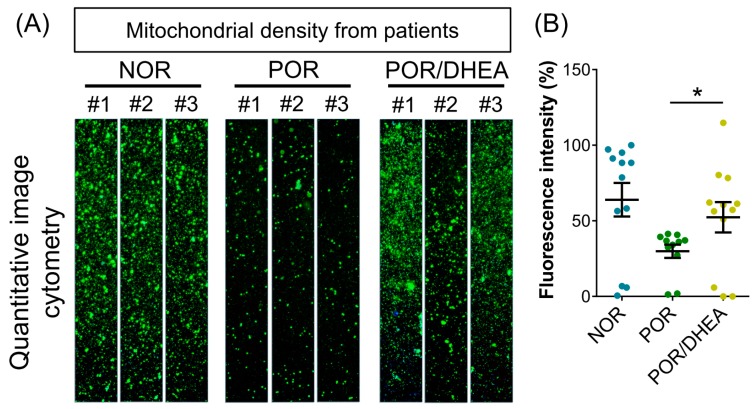
DHEA supplementation improved mitochondrial mass of cumulus cells (CCs) in poor ovarian responders. (**A**) CCs were stained with MitoTracker, and the mitochondrial mass was measured by real-time image cytometry. (**B**) The relative mean of fluorescent intensity was calculated among the eight groups. Data represented the mean ± SEM of nine independent experiments. * *p* < 0.05.

To further assess whether supplementation with DHEA improved mitochondrial function, we analyzed the mitochondrial mass using real-time image cytometry. By visualizing the mitochondria with fluorescent MitoTracker, lower mitochondrial mass was observed in the CCs from the POR group than in those from the NOR group. However, mitochondrial mass in the CCs from the POR group was dramatically elevated following DHEA supplementation (Figure 3). From this finding, it appears that DHEA protects the POR group CCs by improving their cellular mitochondrial mass.

### 3.4. DHEA Restores Mitochondrial Morphology in PORs

**Figure 4:**
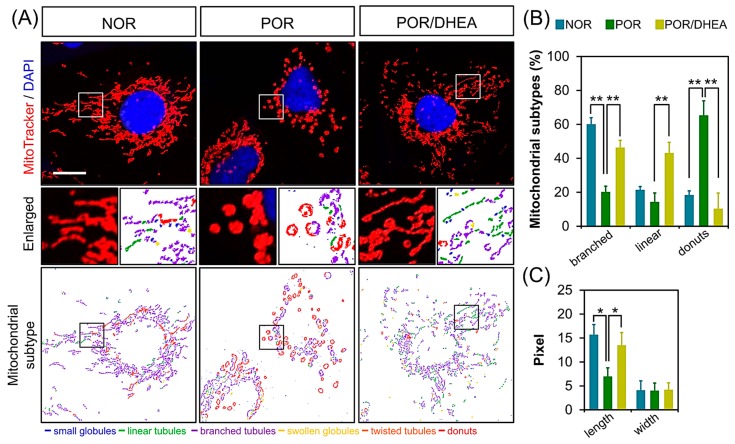
DHEA increased mitochondrial elongation. (**A**) Cumulus cells (CCs) were stained with MitoTracker Red and observed by confocal microscopy to analyze the mitochondrial network structure. MicroP software classifies mitochondria in each cell into three categories according to the features of mitochondrial morphology. (**B**) Three major types of mitochondria were quantified: globular, tubular, and branched, as well as their total length and width. (**B**,**C**) The proportions of different categories of mitochondria obtained from various treatments are shown in (**B**), and the changes in length and width of the mitochondria are shown in (**C**). Data represented the mean ± SEM of 3–5 independent experiments. * *p* < 0.05, ** *p* < 0.01. Scale bar: 20 μm.

To assess whether DHEA treatment was able to protect mitochondria in PORs, we analyzed the mitochondrial network. The mitochondria were classified into three types according to their morphological characteristics (branched, linear, and donuts), using MicroP software [22]. After visualizing the mitochondria with fluorescent MitoTracker, we found that mitochondrial elongation was significantly reduced in the POR group, with mitochondria displaying a donut-like pattern (Figure 4A). Notably, DHEA treatment enhanced the branched type of mitochondria. Our data showed that the POR/DHEA group displayed a significantly higher percentage of branched and linear mitochondria than the POR group. In addition, we also noted that the average length of the mitochondria in the POR/DHEA group was significantly greater than in the untreated groups. The width of the mitochondria did not differ among the three groups (Figure 4B).

### 3.5. DHEA Supplementation Regulates Mitochondrial Dynamics in CCs 

**Figure 5:**
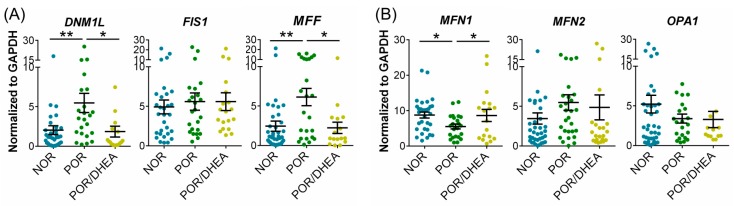
DHEA supplementation regulated mitochondrial dynamics of cumulus cells (CCs) from PORs. (**A**,**B**) Quantitative real-time polymerase chain reaction analysis for relative mRNA levels of major mitochondrial dynamics genes of CCs among the normal ovarian responder (NOR) (*n* = 28), poor ovarian responder (POR) (*n* = 19) and POR/DHEA groups (*n* = 19). * *p* < 0.05, ** *p* < 0.01.

To explore the mechanisms underlying the observed reduction in mitochondrial morphological change from the POR group, the relative expression of mitochondrial dynamic genes was analyzed using qRT-PCR. The mRNA levels of *DNM1L* and *MFF* were significantly lower in CCs from the POR/DHEA group than from the POR group (Figure 5A). Furthermore, *MFN1* mRNA was greater in the CCs from the POR/DHEA group than from the POR group. However, there were no significant differences in *MFN2* and *OPA1* expression levels between the POR and POR/DHEA groups (Figure 5B). This result indicates that DHEA significantly reduces mitochondrial fragmentation and increases the proportion of mitochondrial fusion.

### 3.6. DHEA Supplementation Prevents Mitophagy in CCs from PORs

**Figure 6:**
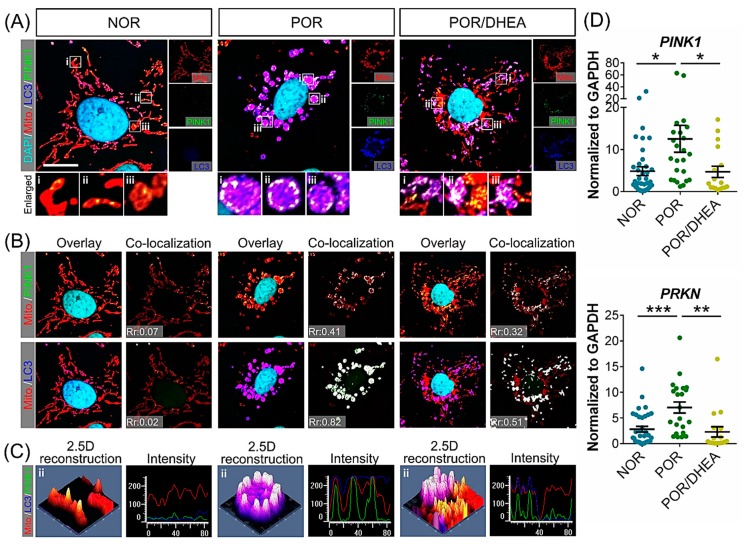
DHEA supplementation decreased mitophagy of cumulus cells (CCs) in poor ovarian responders. (**A**) Confocal images of CCs loaded with MitoTracker, LC3, PINK1, and DAPI. The enlarged images in (**B**,**C**) highlight the representative co-localization with 30× magnification from white squares in the overlay images. (**B**) Co-localization analyses of Mito/PINK1 and Mito/LC3. The co-localization in (**B**) is presented as the product of the differences from the mean (PDM) image. White color pixels indicate co-localization coefficient. (**C**) A 2.5-dimensional reconstruction and fluorescence intensity of the respective insets in (**A**) compared between green, red, and blue fluorescence. Data represented the mean ± SEM of 3–5 independent experiments. (**D**) qRT-PCR analysis for relative mRNA levels of mitophagy genes of CCs (*n* = 19–28). i-iii: enlarged insets * *p* < 0.05, ** *p* < 0.01, *** *p* < 0.001. Scale bar: 20 μm.

To determine the clinical relevance of mitochondrial donuts, we analyzed CCs from patients with and without DHEA supplementation. We investigated the fate of the donut-shaped mitochondria from the POR group. Whole CCs were quadruple-labeled by immunofluorescence and labeled with the autophagosome markers LC3, mitochondria, mitophagy marker PINK1, and DAPI (Figure 6A). In Figure 6B, green fluorescent PINK and red fluorescent mitochondria are indicated, while two fluorescent overlaps appear yellow, while yellow indicated that PINK1 accumulated in defective mitochondria. However, the blue-fluorescent LC3 and red-fluorescent mitochondria overlap in purple, indicating that the mitochondria are coated with autophagosome and enter the autophagy process. Immunofluorescent staining showed that POR increased the level of the autophagosome marker LC3, indicating the activation of autophagy in general. POR also stimulated the co-localization of mitochondria with LC3 and PINK1 (Figure 6B), suggesting a significant reduction in the formation of mitophagosomes and mitochondrial autolysosomes. PINK1 is localized mostly in puncta on LC3, and a reduction of the puncta in the POR group was accompanied by a striped mismatch of mitochondria/PINK1 and mitochondria/LC3. Furthermore, we computed the overall co-localization inside the CCs, and observed that Pearson′s coefficient of the POR/DHEA was significantly reduced from 0.41 to 0.32 compared to the POR group (Figure 6B, upper panel). POR/DHEA displayed reduced docking of PINK1 to mitochondria compared to the POR group (0.82 vs. 0.51; Figure 6B, lower panel). Overall, we have speculated about the change in mitophagy by the color overlap of the images and the Pearson’s coefficient value. The addition of DHEA resulted in a significant decrease of the overlap coefficient between mitochondria/PINK1 and mitochondria/LC3, while PORs with DHEA pretreatment had decreased autophagosome formation. The phenomenon clearly indicated that DHEA reduced mitochondrial damage, which means that DHEA effectively reduces the occurrence of mitophagy to increase the function and quality of mitochondria. We also confirmed the cellular distribution of mitophagy using 2.5-dimensional reconstructions and intensity profiles. In the POR/DHEA group, we further confirmed that the green, red, and blue fluorescence did not fully match that of the POR group (Figure 6C). To explore whether the protective mechanism of DHEA is related to suppression of mitophagy, we assessed the mRNA expression of *PINK1* and *PRKN* by qRT-PCR. The mRNA levels of *PINK1* and *PRKN* significantly decreased in the CCs from the POR/DHEA group compared to those from the POR group (Figure 4D). These results verified the protective effect of DHEA in preventing autophagy associated with the mitochondrial pathway.

## 4. Discussion

In this study, we demonstrated the potential benefit of DHEA supplementation for improving ovarian response to mitochondrial energy stimulation in PORs. To the best of our knowledge, this is the first study to evaluate the effect of DHEA treatment in a specific phenotypic subgroup of PORs. Ovarian reserve and function is an important physiological indicator of female reproductive fitness. The factors negatively affecting ovarian reserve and function are complex, and include advanced age, high body mass index, unhealthy lifestyle, and non-physiological factors, such as ovarian surgery. At present, ovarian reserve decline is increasing, and is positively correlated with the increasing average age of women because of modern increases in lifespan, with approximately 10% of women being infertile [23]. The clinical pregnancy rate is significantly reduced due to the low ovarian reserve. Patients with decreased ovarian reserve and function are difficult groups to assist in achieving pregnancy. Moreover, despite successful pregnancy, the spontaneous abortion rate is relatively high in these patients [22].

DHEA is an androgenic hormone secreted by the adrenal gland, central nervous system, and ovarian follicular cells. It is widely distributed in the tissues and organs of the human body and is converted into more active sex hormones in peripheral tissues [24]. As age increases, secretion of DHEA gradually decreases, as does its level in the circulatory system. When women reach the age of 30, its level drops sharply. Therefore, DHEA may be associated with aging, including ovarian aging. In fact, the mechanism of DHEA in ovarian aging remains unknown. However, increased androgen and insulin-like growth factor-1 (IGF-1) after DHEA supplementation may play key roles. Androgen could increase recruitment and initiation of primordial follicles, development of primary, preantral, and antral follicles, decrease follicular atresia, and increase FSH receptor expression [25,26]. Moreover, IGF-1 is associated with oocyte quality and embryo development [27,28].

Mitochondria exist as a highly dynamic intracellular pool that undergoes constant fission and fusion. The balance of these two processes regulate mitophagy [29]. The fission of mitochondria acts to promote mitophagy, as evidenced by removal of key mediators of fission, such as *FIS1*, *MFF*, or *DNM1L*, leading to an inhibition of mitophagy [30,31]. Mitochondrial fusion is mediated in an opposing manner by diluting damaged mitochondrial components, thereby improving the overall health of the mitochondrial pool and inhibiting mitophagy. Mitochondrial morphology itself can influence the induction of mitophagy. The formation of tubular mitochondrial networks during nutrient deprivation inhibits mitophagy via downregulation of DNM1L [32]. The most studied pathway for mitophagy induction is regulated by PINK1 and Parkin, where PINK1 recruits Parkin to depolarized mitochondria. Once recruited to mitochondria, Parkin ubiquitinates outer mitochondrial membrane proteins for recognition and engulfment by autophagosomes and subsequent degradation in lysosomes. Several Parkin-independent pathways also induce mitophagy. Mitophagy is balanced by mitochondrial biogenesis for generation of new mitochondria [33]. Although one study provided promising insight into the effective mechanisms of DHEA that involve *PRKN* and *PINK1*, fully understanding the roles of DHEA on the functional activation of *PRKN* and *PINK1* in different pathological processes still needs to be accomplished. Promisingly, Figure 6B shows that DHEA significantly reduced PINK1 mitochondrial translocation in CCs from POR. Furthermore, DHEA downregulated the formation of LC3 and decreased the level of *PRKN* and *PINK1*, suggesting that it affects LC3-mediated mitophagy.

In addition to the appearance evaluation to assess problems with mitochondria, we can detect molecular mechanism through known markers of fusion and fission. Many genes are involved in the molecular regulation of mitochondria morphology changes. We analyzed the expression of OPA1, which regulates mitochondrial inner membrane fusion, and the genes of two mitochondrial outer membrane proteins, MFN1 and MFN2, because the division and integration are dynamic and complementary. We then analyzed three fission genes to confirm the results. DHEA significantly increased the expression of the MFN1 fusion gene. Interestingly, although DHEA has hardly improved the fusion gene, the fission genes DNM1L and MFF were significantly reduced. This also shows that DHEA supplementation not only effectively slows down the formation of the donut shape in mitochondria of CCs, but also confirms that the performance of the fission gene is greatly reduced at the molecular level. In the future, we will increase the number of samples to improve the accuracy of the experiment.

Our previous study found that the expression of TFAM gene, mitochondrial dehydrogenase activity, and mitochondrial mass, were higher in the CCs from the POR/DHEA group than those from the POR group. TFAM is an essential protein that binds mitochondrial DNA (mtDNA) to regulate mitochondrial transcription initiation and is also a key regulator of mtDNA copy number [34]. The abundance of mtDNA generally reflects TFAM levels [34]. The results suggested that DHEA supplementation had the positive effects on the mitochondrial function in CCs; several studies using cell culture or an animal model also supported the beneficial effects of DHEA treatment on the mitochondrial function [35,36,37,38]. In a study using human keratinocytes, DHEA reversed serum deprivation-induced reduction of mitochondrial membrane potential to basal levels and conserved mitochondrial membrane integrity [35]. Furthermore, DHEA significantly increased the activities of superoxide dismutase, catalase, and peroxidase, and decreased the loss of mitochondrial membrane potential and the level of reactive oxygen species in the Leydig cells [36]. Data obtained in previous studies using rat models have demonstrated that treatment with DHEA could ameliorate oxidative energy metabolism via the stimulation of ATPase activity, mitochondrial dehydrogenase activities and oxygen consumption rate (OCR) in the mitochondria [37,38,39,40]. Although, many studies have indicated that DHEA has the function of regulating mitochondria. However, there are no specific reports indicating that DHEA specifically regulates targets in mitochondria. The future studies addressing the affinity and time/tissue-dependent downstream effects of DHEA are warranted to fully understand the role of this sterol and its sulfated form in mitochondrial function.

CCs are the somatic cells that surround the oocytes and form the COC with oocytes [41]. The COC communicates bidirectionally via specialized gap junction and paracrine signaling factors [41]. CCs could protect and nurture oocytes [42] and play an important role in oocyte maturation, oocyte meiosis, ovulation, and fertilization [43,44,45]. In addition, oocytes could regulate CC functions, including proliferation, apoptosis, luteinization, metabolism, and expansion via oocyte-secreted factors [46]. Oocytes direct the surrounding somatic cells to conduct functions required for oocyte development. Therefore, CCs and oocytes have a close relationship and affect each other. Several studies have suggested that CC gene expression can serve as biomarkers for oocyte competence [13,47,48]. Hence, CCs could be considered as an alternative representative of oocytes. Mitochondria of CCs are involved in oocyte maturation, embryo development, and pregnancy prognosis [49,50]. An observational study conducted by Boucret and colleagues suggested that mitochondrial features of the CCs could serve as indicators of oocyte competence [49]. In the study of Ogino et al. the determination of mtDNA content in CCs can be used to predict good-quality embryos [50]. Tsai and colleagues performed a prospective cohort study that revealed lower levels of mitochondria deletion in CCs were associated with higher pregnancy rates in IVF cycles [51]. Therefore, enhancing mitochondrial biogenesis may result in an improvement of oocyte and embryo quality and, subsequently, better pregnancy outcomes because increased mitochondrial energy production may decrease chromosomal aneuploidies, improve embryo cleavage, and reduce cytoplasmic fragmentation [52]. Our previous studies showed that DHEA could increase mitochondrial function and activity, and reduce apoptosis and cell necroptosis in CCs and human granulosa cell line [3,4,53]. In addition, we found that DHEA might slow the aging of CCs [17]. This study proposes a new concept: in addition to increasing the mitochondrial mass, DHEA can also reduce mitochondrial fission and increase clearance of poorly functioning mitochondria by mitophagy. DHEA effectively improves the function and quality of cumulus cell mitochondria in PORs.

An important limitation of our study was its small sample size. Therefore, a trend of increase was observed in clinical outcomes, but it was not significant. Additionally, although the age was not significantly different between POR and POR/DHEA groups, the age of the POR group was higher than that of the POR/DHEA group. Thus, we should interpret the data carefully.

## 5. Conclusions

In conclusion, this study showed that DHEA supplementation increased mitochondrial mass, decreased mitochondrial fission, and enhanced the clearance of dysfunctional mitochondria via mitophagy. Our observations may provide a possible rationale for the clinical use of DHEA supplementation in PORs undergoing IVF cycles.

## Figures and Tables

**Table 1:** Basic characteristics of patients in the NOR, POR, and POR/DHEA groups.

Parameters	NOR (*n* = 28)	POR (*n* = 19)	POR/DHEA (*n* = 19)
Age (years)	36.2 ± 3.0	40.5 ± 4.3 *	37.8 ± 3.7
Body mass index (kg/m^2^)	24.2 ± 3.8	22.1 ± 3.9	22.5 ± 3.4
Duration of infertility (years)	3.8 ± 2.6	3.5 ± 2.9	6.0 ± 4.5
Previous IVF failure (*n*)	1.1 ± 1.1	1.7 ± 2.2	3.5 ± 2.6 *^#^
Types of infertility (%)			
Primary infertility	12/28 (43%)	9/19 (37%)	10/19 (52%)
Secondary infertility	16/28 (57%)	12/19 (63%)	9/19 (47%)
Basal FSH (IU/L)	4.3 ± 1.5	6.1 ± 5.3	6.1 ± 3.5
Basal E2 (pg/mL)	94.9 ± 90.8	105.6 ± 72.7	99.2 ± 78.4
Basal LH (IU/L)	4.4 ± 2.2	4.0 ± 5.3	6.3 ± 11.1

NOR, normal ovarian responder; POR, poor ovarian responder; DHEA, dehydroepiandrosterone; IVF, in vitro fertilization; FSH, follicle stimulation hormone; E2; estradiol; LH, luteinizing hormone. * *p* < 0.05 versus NOR; # *p* < 0.05 versus POR.

**Table 2:** Cycle characteristics and pregnancy outcome in the NOR, POR, and POR/DHEA groups.

Parameters	NOR (*n* = 28)	POR (*n* = 19)	POR/DHEA (*n* = 19)
Stimulation duration (days)	10.9 ± 1.8	10.4 ± 2.2	10.6 ± 1.5
HMG/FSH dose (IU)	3190.3 ± 720.1	2775.3 ± 857.5	2992.1 ± 577.7
No. of oocytes retrieved (n)	10.7 ± 5.1	3.0 ± 1.9 *	4.1 ± 3.0 *
No. of metaphase II oocytes (n)	6.6 ± 3.9	1.6 ± 1.5 *	2.0 ± 1.3 *
Maturation rate (%)	60.3 ± 20.1	47.4 ± 35.0	65.5 ±3 2.3
No. of fertilized oocytes (n)	7.3 ± 3.4	2.3 ± 1.7 *	2.6 ± 1.6 *
Fertilization rate (%)	69.3 ± 15.6	69.8 ± 31.8	75.5 ± 22.0
No. of day 3 embryos (n)	6.2 ± 3.0	2.2 ± 1.6 *	2.2 ± 1.4 *
No. of top-quality D3 embryos (n)	2.6 ± 2.2	0.8 ± 1.3 *	0.8 ± 1.0 *
Clinical pregnancy rate % (n)	50.0% (14/28)	11.1% (2/18) *	26.3% (5/19)
Ongoing pregnancy rate % (n)	42.9% (12/28)	11.1% (2/18) *	26.3% (5/19)
Live birth rate % (n)	42.9% (12/28)	11.1% (2/18) *	16.7% (3/18)

NOR: normal ovarian responder; POR: poor ovarian responder; DHEA: dehydroepiandrosterone; HMG: human menopausal gonadotrophin; FSH: follicle stimulation hormone. * *p* < 0.05 versus NOR.

## Supplementary Materials

The following are available online at http://www.mdpi.com/2077-0383/7/10/293/s1, Table S1: Sequence of oligo-nucleotides used as RT-PCR primers.
