# Supplementary material for: Dehydroepiandrosterone Ameliorates Abnormal Mitochondrial Dynamics and Mitophagy of Cumulus Cells in Poor Ovarian Responders
Source: J Clin Med. 2018 Sep 20;7(10):293. doi: 10.3390/jcm7100293 (PMC6210273; doi:10.3390/jcm7100293)
Supplement: Supplementary file 1 [file jcm-07-00293-s001.pdf]

Table S1. Sequence of oligo-nucleotides used as RT-PCR primers.

Gene	Primers	Genbank accession number
GAPDH	Forward primer: CGACCACTTTGTCAAGCTCA	NM_002046
	Reverse primer: AGGGGAGATTCAGTGTGGTG	
MFN1	Forward primer: TGTTTTGGTCGCAAACTCTG	NM_033540
	Reverse primer: TCTTTCCATGTGCTGTCTGC	
MFN2	Forward primer: TGTTGGCTCAGTGCTTCATC	NM_001127660
	Reverse primer: AAGTCCCTCCTTGTCCCAGT	
OPA1	Forward primer: GGCCAGCAAGATTAGCTACG	NM_001354663
	Reverse primer: CACAATGTCAGGCACAATCC	
Drp1	Forward primer: ACCCGGAGACCTCTCATTCT	NM_001278463
	Reverse primer: TTGACAACGTTGGGTGAAAA	
FIS1	Forward primer: CTTGCTGTGTCCAAGTCCAA	NM_016068
	Reverse primer: GGCTGAAGGACGAATCTCAG	
PINK	Forward primer: AGATGAGGCTGGACTGAGGA	NM_032409
	Reverse primer: TCCCACTCCCGTAACTGAAC	
PARKIN	Forward primer: GCATCTTCCAGCTCAAGGAG	NM_004562
	Reverse primer: CTTTTCTCCACGGTCTCTGC	
MFF	Forward primer: GATCAGATTCTGCCCCAAGA	NM_001277061
	Reverse primer: TTTTCATCCAGCACATCCAA	
